# A novel fully human anti-ROR1 antibody PBA-0405 optimized for ADCC, induces potent anti-tumor activity against both solid and hematological malignancies

**DOI:** 10.3389/fimmu.2025.1711509

**Published:** 2025-12-17

**Authors:** Niina Veitonmäki, Anna Kostyn, Sonia Kołt, Aleksandra Ziemblicka, Beata Romanowska, Sabina Oleksy, Ewelina Krzywińska, Kamilla Sołtys, Łukasz Świderski, Piotr Naporowski, Piotr Mamczur, Monika Świtoń, Marek Orłowski, Ying Zhang, Jianming Xu, Anna Andrzejczak, Aleksandra Bielawska-Pohl, Lidia Karabon, Dariusz Wołowiec, Wondossen Sime, Ramin Massoumi, Sameer Deshmukh, Jakub Kołodziejski, Tomasz Klaus, Pieter Spee

**Affiliations:** 1 Pure Biologics S. A., Wroclaw, Poland; 2 Department of Biochemistry, Molecular Biology and Biotechnology, Faculty of Chemistry, Wroclaw University of Science and Technology, Wroclaw, Poland; 3 GemPharmatech Co. Ltd., Nanjing, China; 4 Laboratory of Genetics and Epigenetics of Human Diseases, Hirszfeld Institute of Immunology and Experimental Therapy, Polish Academy of Sciences, Wroclaw, Poland; 5 Laboratory of Biology of Stem and Neoplastic Cells, Hirszfeld Institute of Immunology and Experimental Therapy, Polish Academy of Sciences, Wroclaw, Poland; 6 Clinical Department of Hematology, Cell Therapies and Internal Diseases, Wroclaw Medical University, Wrocław, Poland; 7 IVRS AB, Lund, Sweden

**Keywords:** ROR1, antibody, ADCC, afucosylation, cancer, leukemia, CLL, PBA-0405

## Abstract

**Introduction:**

B-cell malignancies, including chronic lymphocytic leukemia (CLL), pose a significant clinical challenge due to high relapse rates and dose-limiting toxicities seen with current therapies, particularly in elderly patients. Receptor tyrosine kinase-like orphan receptor 1 (ROR1) is an attractive therapeutic target due to its high expression on malignant B-cells and minimal presence in normal adult tissues, allowing the development of more potent and safer drugs for cancer treatment. PBA-0405 (called 2.0405.aF in this manuscript) is a novel, fully human afucosylated anti-ROR1 monoclonal antibody, specifically engineered for optimal induction of antibody-dependent cellular cytotoxicity (ADCC) to improve treatment of cancer.

**Methods:**

We evaluated 22.0405.aF for ADCC activity and tumor-cell killing in comparison with clinically tested anti-ROR1 antibodies, including zilovertamab and R12. Functional assessment included *in vitro* cytotoxicity assays, *ex vivo* killing of patient-derived malignant B cells, and in vivo testing in humanized mice and murine xenograft models of hematologic and solid tumors. Safety was assessed through monitoring of healthy B-cell viability and toxicity *in vivo*.

**Results and discussion:**

Our findings demonstrate that 22.0405.aF exhibits far superior ADCC activity compared to anti-ROR1 antibodies, e.g. zilovertamab and R12, tested in clinical trials thus far. 22.0405.aF displayed potent tumor cell killing, both *in vitro* and *in vivo*, and induced more potent killing of patient-derived malignant B-cells *ex vivo* compared to rituximab, without affecting healthy B-cells. Furthermore, in humanized mice, in murine xenograft models of both hematological and solid tumors, 22.0405.aF treatment induced robust inhibition of tumor growth without detectable toxicity.

**Conclusion:**

These results underscore the therapeutic potential of 22.0405.aF as a promising immunotherapy for treatment of ROR1-positive B-cell malignancies. Notably, 22.0405.aF has recently completed Phase 0 clinical trials in patients with head and neck carcinomas and soft tissue sarcomas, paving the way for further clinical development.

## Introduction

Chronic lymphocytic leukemia (CLL) is the most common hematologic malignancy in the Western world and is considered incurable (1). Both CLL and mantle cell lymphoma (MCL) are associated with disabling symptoms including lymphadenopathy and organomegaly, which can lead to life-threatening complications including organ failure, myelosuppression, and immunocompromise with increased risk for bleeding and infections (2, 3). While current treatments such as Bruton’s tyrosine kinase (BTK) and B-cell lymphoma 2 (BCL-2) inhibitors, as well as chemoimmunotherapy regimens (e.g., fludarabine or cyclophosphamide in combination with rituximab or obinutuzumab), have significantly improved response rates and overall survival, eventual relapse and intolerance to therapy remains inevitable in B-CLL treatment (4–6). Moreover, many patients, particularly elderly individuals or those with comorbidities or compromised bone marrow functions, are unable to tolerate the toxicities associated with these therapies. CD20-targeting monoclonal antibodies like rituximab and obinutuzumab efficiently induce tumor cell killing, to the extent of causing tumor lysis syndrome (TLS), but can also lead to severe infections and reactivation of viruses such as hepatitis B, partly due to the elimination of healthy B cells by these antibodies (7–10). BTK inhibitors, including ibrutinib, acalabrutinib, and zanubrutinib, carry risks of serious cardiovascular toxicities such as atrial fibrillation and ventricular arrhythmias (11, 12). Because of these challenges, only 5% of patients are treated at diagnosis, and nearly 70% require intervention only after disease progression (13). Thus, there is a critical need for therapies that provide long-term remission with fewer adverse effects to enable earlier intervention.

A promising approach to reduce treatment-related toxicity in drug development involves pursuing drug targets only expressed on tumor cells. The oncofetal receptor tyrosine kinase-like orphan receptor 1 (ROR1) is selectively expressed on malignant cells, including in over 90% of B-CLL cases, while being nearly absent in normal adult tissues. The therapeutic potential targeting ROR1 in B-CLL tumor cell survival and proliferation has been validated in clinical trials. Zilovertamab (cirmtuzumab, UC-961), an anti-ROR1 monoclonal antibody developed as a ROR1 antagonist, has shown encouraging therapeutic effects in patients with B-CLL and MCL with a good safety profile (14, 15). Here, we introduce PBA-0405 (22.0405.aF, afucosylated form of 22.0405), a novel fully human afucosylated anti-ROR1 IgG1 antibody design that combines the advantages of ROR1 targeting observed in zilovertamab clinical development with the proven therapeutic principle of killing malignant B cells through antibody-dependent cellular cytotoxicity (ADCC), as pioneered by rituximab. We describe the development, characterization, and preclinical validation of 22.0405.aF, a potential first-in-class afucosylated anti-ROR1 antibody, which recently completed Phase 0 clinical investigation (NCT06273852) in patients with head and neck carcinomas and soft tissue sarcomas.

## Materials and methods

### Cells

Raji cells (CCL-86) were purchased from ATCC, JeKo-1 (ACC 553) and MEC-1 (ACC 497) were obtained from DSMZ, and CHOK1 (#85051005) was acquired from ECACC. Raji, JeKo-1, and MEC-1 were cultured in RPMI 1640 medium (Gibco), and CHO cells were cultured in DEM/F12 medium. All cells were supplemented with 10% fetal bovine serum (Sigma-Aldrich) (JeKo-1 20%), 1%–2% L-glutamine (Gibco), and 1% penicillin/streptomycin (PEST, GIBCO). All cell lines were kept at 37°C and 5% CO_2_ and cultured for less than 20 passages. Raji, CHO, or MEC-1 cells were transfected to stably express ROR1 and were cultivated with puromycin for pressure selection. Primary CLL material were obtained from the Clinical Department of Hematology, Cell Therapies and Internal Diseases, Wroclaw Medical University, Wrocław, Poland via the Laboratory of Genetics and Epigenetics of Human Diseases, Hirszfeld Institute of Immunology and Experimental Therapy, Hirszfeld Institute, Wroclaw, Poland in accordance with ethical approval from the local Bioethical Committee at the Medical University of Wroclaw (KB-467/2016), Poland.

### Antibody generation

Phage display selections were conducted using a fully human Ancestral scFv (single-chain variable fragment) and scFv Hyperimmune Library (Twist Biosciences) against different domains of human ROR1, including the Ig-like, the Frizzled, and the Kringle domain, as well as against the full-length extracellular portion of human ROR1. Phage pannings were performed using recombinant proteins and a combination of recombinant proteins and whole cells. The most promising sequences from the selected phage pools were expressed as scFvs and tested for ROR1 binding in cell-based assays using flow cytometry. Specificity of the ROR1-binding scFv was validated by counter-screening against cells transfected with empty vectors or ROR2-transfected cells. The top antibody sequences were then expressed both as native IgG1 antibodies and as afucosylated IgG1 antibodies in CHOK1 cells (BioIntron, China) and GlyMaxX CHO cells (Evitria, Switzerland), respectively. Reference antibodies zilovertamab and R12-like antibodies were generated based on sequences available from databases, patents, and publications (16, 17). The 22.0405.aF sequence is described in the patent WO2024251787A1. Isotype controls were generated using phage display library selections against streptavidin, and antibodies were expressed in both native and afucosylated formats in CHOK1 cells (BioIntron, China) and GlyMaxX CHO cells (Evitria, Switzerland), respectively. The antibodies are referred to as Ctr IgG1 and IgG1.aF, respectively.

### Cell-based binding assays

Cells overexpressing either ROR1 or ROR2 or transfected with empty vector, or cells expressing ROR1 endogenously were incubated with different concentrations of CSFE CellTrace™, at room temperature (RT), for 30 min. Washed cells were incubated with different concentrations of ROR1 antibodies for 30 min on ice followed by washing. After incubation with allophycocyanin (APC)-labeled AffiniPure Goat Anti-Human IgG Fcγ fragment-specific antibody (Jackson ImmunoResearch) for 30 min on ice, washed cells were stained with Sytox Blue (Invitrogen) and analyzed by flow cytometry using an Attune™ NxT Focusing Cytometer equipped with a CytKick autosampler (Thermo Fisher Scientific).

### Molecular binding assays using BLI

The apparent affinity of antibodies was determined using Octet RED384 (Sartorius). Antibodies were captured on FAB2G (Anti-hIgG CH1 Capture) biosensors (Sartorius) at 5 μg/mL (34 nM). Twofold serial dilution of recombinant human ROR1/Fc, human ROR2/Fc, mouse ROR1/Fc (R&D Systems) and mouse ROR2/Fc (Acro Biosystems) (200–3.13 nM) was prepared in 10× Kinetics Buffer (Sartorius). Analyte associations were measured for 200 s followed by 500 s dissociation in 10× Kinetics Buffer. Biosensors were regenerated after each binding cycle with 10 mM glycine, pH 1.7 (Cytiva). Sensorgrams were referenced by blank (buffer) subtraction, and the association (*k*_on_) and dissociation (*k*_off_) rate constants were evaluated from global fitting based on a 1:2 (bivalent analyte) binding model using Octet Analysis Studio 12.2 software (Sartorius). The apparent dissociation constant was calculated from the equation: *K*_D_ = *k*_off1_/*k*_on1._

### Molecular binding assays using SPR

The antibody affinities were determined using surface plasmon resonance (SPR) with a Biacore 8K instrument (Cytiva). Recombinant FcγR proteins (R&D Systems) were biotinylated and immobilized on the CAP chips (Cytiva), according to the supplier’s instruction. Serial dilutions of antibodies as analytes were prepared in PBS-P+ buffer (Cytiva) and injected at a flow rate of 30 μL/min. Data were double-reference subtracted (reference cell and blank subtraction) and analyzed using Biacore™ Insight Software (Cytiva). Association (*k*_on_) and dissociation (*k*_off_) rate constants were evaluated based on a 1:1 Langmuir binding model and equilibrium dissociation constants were calculated from the equation: *K*_D_ = *k*_off_/*k*_on._

### Epitope binning

Epitope binning was performed using the Octet RED384 instrument (Sartorius). Antibodies [5 µg/mL (34 nM) in 10 mM acetate buffer, pH 6.0, Cytiva] were immobilized on AR2G sensors. The resulting antibody sensors were incubated in 1,000 nM ROR1-His (Acro Biosystems, 100 s) followed by 1,000 nM ROR1-His premixed with 500 nM of competing antibodies for 100 s, and dissociation was measured for 200 s. For binding studies to different domains of ROR1, antibody-coated ARG2 sensors were dipped in samples of 500 nM recombinant Ig-like, Frizzled, or Kringle (Acro Biosystems) for 150 s, followed by a dissociation step in buffer for 200 s. Sensorgrams were evaluated with the Octet Analysis Studio 12.2 software (Sartorius).

### ADCC

Peripheral blood mononuclear cells (PBMCs) were obtained from healthy donor buffy coat samples, in agreement with the local Wroclaw blood donation station. Density centrifugation was performed with SepMate™-50 PBMC Isolation Tubes and Lymphoprep (Stemcell™) followed by lysing red blood cells with Red Blood Cell Lysis Buffer (BioLegend). Natural killer (NK) cells were isolated from PBMCs by immunomagnetic negative selection with the NK Cell Isolation Kit (Miltenyi Biotec) according to the manufacturer’s protocol. NK cells were characterized by purity and phenotype.

Target cells were labeled with 0.1 µM of the CellTrace™ CFSE Proliferation Kit (Invitrogen™), seeded into a conical bottom 96-well plate (Thermo Scientific™, Nunc™) together with serial concentrations of antibodies. NK cells were added to each well at a 10:1 ratio of effector cells to target cells. After 4 h of incubation at 37°C, the cells were stained with Live/Dead™ Fixable Violet Dead Cell Stain Kit solution (Invitrogen™), fixed with 4% paraformaldehyde (Thermo Scientific™), and subjected to flow cytometry. Data were analyzed using FlowJo™ v10.8.1 software (BD Life Sciences) and GraphPad Prism 7.05 software (GraphPad Software, Inc.). In combination ADCC, venetoclax or ibrutinib at different concentrations was incubated with target cells overnight before the ADCC assay and analyzed by flow cytometry. Live imaging of ADCC using NK cells and JeKo-1 was performed using Calcein AM (Thermo Fischer Scientific) for cell staining and the Opera Phenix High Content Screening System (Revity) for imaging.

### CD107a degranulation assay and measurement of cytokine release

Fresh PBMCs were purified from healthy donor buffy coats from Wrocław blood donor center and incubated overnight at 37°C in IMDM (Gibco™, #12440053) supplemented with 10% FBS and 55 μM of β-mercaptoethanol (Gibco™, #21985023). JeKo-1 cells were preincubated for 30 min at 37°C with 50 nM of either 22.0405, 22.0405.aF, zilovertamab, R12 hIgG1, or rituximab. Subsequently, target cells were washed and incubated with PBMCs, for 4 h at 37°C at an effector:target ratio of 1:1, in the presence of APC-conjugated human CD107a antibody (BioLegend, Clone: H4A3, Cat #328620) and protein transport inhibitors (BD GolgiStop, Cat #554724 and BD GolgiPlug, Cat #555029). Subsequently, cells were incubated with blocking solution, then labeled with aqua viability dye (LIVE/DEAD™ Fixable Aqua Dead Cell Stain Kit, Cat #L34957), BV605 anti-human CD56 (BD Biosciences, Clone: NCAM16.2, Cat #562779), BV421 anti-human CD16 (BioLegend, Clone: 3G8, Cat #302038), and APC-H7 anti-human CD3 (BD Biosciences, Clone: SK7, Cat #560176), fixed with BD Cytofix/Cytoperm™ (BD Biosciences, Cat #554714), followed by intracellular staining for BB700 anti-human interferon-γ (IFN-γ) (BD Biosciences, Clone: B27, Cat #566394) and BV750 anti-human tumor necrosis factor-α (TNF-α) (BD Biosciences, Clone: Mab11, Cat #566359). Isotype-matched antibodies from the same manufacturer (BD Biosciences or BioLegend) were used to assess background fluorescence. Cells were run through a FACS Cytek^®^ flow cytometer (Cytek Biosciences) with standard equipment. Data were analyzed using FlowJo v10.

### ADCP

Monocytes were enriched from frozen PBMCs using the human Pan Monocyte Isolation Kit (Miltenyi Biotec, #130-096-537) according to the manufacturer’s instructions. Purification was verified by phenotypic analysis of surface markers: PE anti-human CD14 (BioLegend, Clone: M5E2, Cat #982508) and Purification was verified by phenotypic analysis of surface markers: APC anti-human CD16 (BioLegend, Clone: 3G8, Cat #980104). The enriched monocyte fractions from PBMCs were cultured for 7 days in IMDM (Gibco™, Cat #12440053) and 10% FBS, in the presence of 50 ng/mL M-CSF (BioLegend, Cat #574804) + 50 ng/mL IL-4 (BioLegend, Cat #766204) + 10% human serum. The adherent population of differentiated macrophages was stained with 0.1 µM Violet Dye (Invitrogen, CellTrace™ Violet Cell Proliferation Kit, Cat #C34557). Differentiated macrophages were verified with stainings for CD163 (BV421 anti-human CD163 BD Biosciences, Clone: GHI/61, Cat #562643), CD16 (APC anti-human CD16 BioLegend, Clone: 3G8, Cat #980104), CD32 (FITC anti-human CD32 BioLegend, Clone: FUN-2, Cat #303204), and CD64 (PE anti-human CD64 BioLegend, Clone: 10.1, Cat #983202) using flow cytometry.

Before the experiment, JeKo-1 cells were stained with 0.1 μM CFSE Dye (CellTrace™ CFSE Cell Proliferation Kit, Invitrogen™, #C34570) and preincubated with 50 nM of the respective 22.0405.aF, R12, zilovertamab, rituximab, or isotype control antibodies. The Violet Dye labeled macrophages were co-cultured with CFSE-labeled JeKo-1 cells and incubated at a 2:1 effector: target ratio for 4 h, at 37°C. Cells were subsequently stained with viability dye [LIVE/DEAD Fixable Near-IR (780) Viability Kit, Invitrogen™, #L34994] and analyzed using FACS Attune (Thermo Fisher Scientific) to measure phagocytosis. Percentage of cell phagocytosis was calculated using the formula: number of dual stain-positive target cells (cells engulfed by macrophages; Violet+/CFSE+) divided by the total number of target cells (CFSE+). Data were analyzed using FlowJo v10.

### C1q binding

MaxiSorp ELISA (enzyme-linked immunosorbent assay) 96-well plates (Thermo Fisher Scientific) were coated with either 22.0405.aF, rituximab, or isotype control at 5 µg/mL (34 nM) overnight at +4°C. Washed plates were blocked with 2% bovine serum albumin (BSA) in PBS for 1 h at RT followed by washing, and serially diluted recombinant C1q protein (Sigma-Aldrich, #204876) was transferred into the wells. The detection was performed with anti-C1q-HRP antibodies (Invitrogen™, #PA1-84324). Upon adding TMB substrate (BD OptEIA™, #555214), the plates were read at 450- and 650-nm wavelength using Microplate spectrophotometer SPARK 10M with luminescence (TECAN).

### huHSC-NCG-hIL15 JeKo-1 model

The experiments were performed at GemPharmatec according to the local ethical permissions. Fifty-six (+ surplus animals) huHSC-NCG-hIL15 female mice (GemPharmatech, China) were subcutaneously injected with JeKo-1 cells resuspended in DPBS. The huHSC-NCG-hIL15 model was generated by reconstitution of CD34^+^ huHSC from independent donors in irradiated NCG-hIL15 mice, and reconstitution level was confirmed by flow cytometry as >25% hCD45^+^ of total living cells in the peripheral blood. When the average tumor size reached approximately 82 mm^3^, mice were randomly grouped and treated biweekly with intravenous injections of 22.0405.aF or vehicle at 10 mg/kg. Tumors were measured twice weekly, and blood was sampled at 4 and 24 h after the first treatment. The mice were inspected daily. At the endpoint, tumors and spleens were collected, weighted, and cut in half. One-half was processed into a single-cell suspension. After cell counting, an aliquot of 3 × 10^6^ cells was used for antibody staining together with red blood cell lysed terminal blood and analyzed in flow cytometry for cell markers: eBioscience™ Fixable Viability Dye eFluor™ 506, mCD45-BV605, hCD45-PerCP-Cy5.5, hCD3-FITC, hCD56-APC, hCD69-PED594, hCD107a, BC785 hGranzyme B-PE-Cy7, hROR1-PE (#357804) (BioLegend), hCD8-BV650, hCD25-BV711, and hIFN-γ -BV421 (BD Biosciences). The number of cells analyzed by flow cytometry was normalized by 0.1 g of the original organ/tumor weight to avoid weight-based differences. Gating strategy is shown in **Supplementary Figure 4**.

The other half of the tumors were fixed with 4% PFA and processed for immunohistochemistry. The tumor slides were stained for ROR1 (Thermo Fisher Scientific, PA5-14726), CC3, CD56, Granzyme-B, CD68 (Cell Signaling Technology #9664S, #99746, # 46890, and #76437, respectively), CD16 (Protein Tech, #16559-1-AP), and IFN-γ (Abcam, #231036), and the slides were analyzed by the Vectra M3 3D Imaging system (3D).

### MEC-1-ROR1 model

The experiment was performed at IVRS, Lund, Sweden, under Lund/Malmo ethical approval. CB-17/Icr-Prkdcscid/scid/Rj immunodeficient female mice from Janvier, France, were injected intravenously with MEC-1-ROR1 cells (1 × 10^6^) and treatments (six in total at 10 mg/kg) were given twice weekly after 1 week of tumor cell inoculation. After 4 weeks of tumor cell inoculation, the spleen and bone marrows from femur and tibia of both hind legs were isolated from each transplanted mouse. Flow cytometry analysis was performed on splenic and bone marrow-derived cells using anti-human ROR1-PE (R&D Systems, #FAB2000P) and anti-human CD19-APC (BD, #HIB19) antibodies, as well as the corresponding isotype controls. Cells were subjected to data acquisition and multiparametric FACS assessment by using a four-laser BD FACSMelody™ instrument (BD Biosciences). Data were analyzed using FlowJo software, and both the mean and median readout were recorded for CD19^+^ or ROR1 cells after the background was set using the respective isotype controls.

### Statistics

Statistics were calculated using GraphPad Prism version 7.05 or 9 using one-way analysis of variance (ANOVA) (Kruskal–Wallis multiple comparison) or *in vitro* dose responses using two-way ANOVA with Tukey’s or Sidak’s multiple comparison, according to GraphPad Prism analysis recommendation. For single comparisons, Mann–Whitney non-parametric analysis was performed. Tumor growth curves were considered statistically significant using two-way ANOVA, with Bonferroni multiple comparison. All statistics are two-tailed *p*-values if not specified otherwise.

## Results

### Native 22.0405 antibody binds to a differential epitope compared to a zilovertamab-like antibody leading to a potent ADCC induction

**Figure 1:**
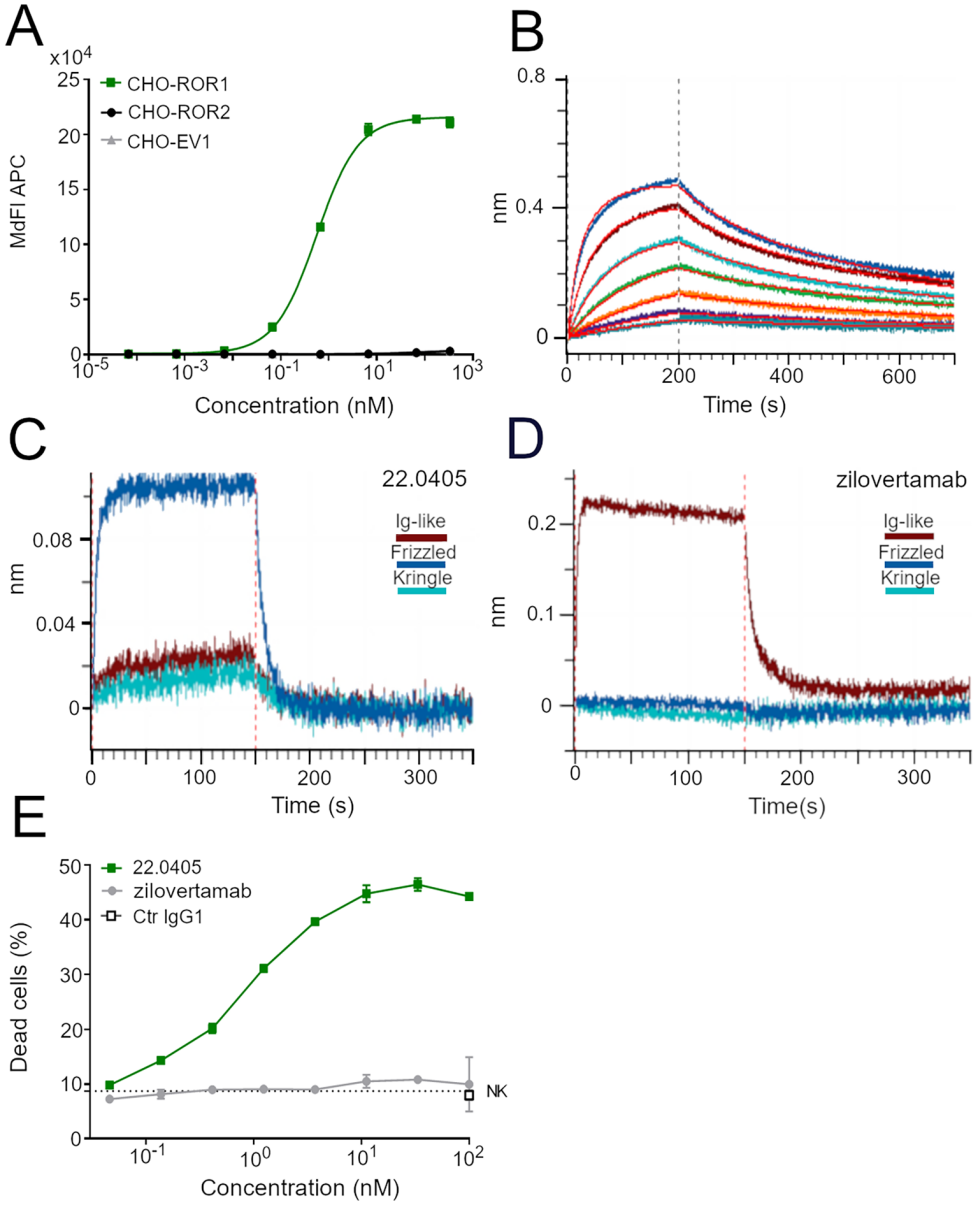
Binding properties of 22.0405 to ROR1. **(A)** Different concentrations of 22.0405 were titrated on CHO cells overexpressing ROR1, ROR2, or the empty vector (EV) control cell line, and the binding potency was analyzed by flow cytometry. The graph shows the mean ± standard deviation (SD) of a representative experiment performed three times in duplicates. **(B)** BLI binding analysis or 22.0405 to recombinant extracellular part of ROR1 at doses between 3.31 and 200 nM. The graph is a representative experiment from at least five different experiments performed in triplicates. **(C, D)** BLI binding analysis of 22.0405 or zilovertamab-like antibody against 500 nM of recombinant Frizzled, Kringle, or IgG1 domain of hROR1. **(E)** NK cell-mediated ADCC against JeKo-1 cells after 4h incubation analyzed by flow cytometry. Graph shows mean ± SD of triplicates; the dashed line indicates NK-mediated killing in the absence of antibody. The graph is a representative figure of minimally three different donors performed in duplicates or triplicates.

Using phage display technology, a fully human native ROR1-specific IgG1 mAb was generated, named 22.0405, with a strong capacity to induce NK-mediated ADCC against ROR1-expressing cells. The specificity of 22.0405 for ROR1 was confirmed in molecular and cellular binding assays. Using CHO-K1 cells transfected with either human *ROR1*, *ROR2*, or empty vector, 22.0405 bound to CHO-ROR1 cells with an EC_50_ of 0.56 nM (**Figure 1A**, **Table 1**) but did not bind to CHO-ROR2 or empty vector-transfected cells. For comparison, an anti-ROR1 mAb mimicking zilovertamab, a humanized IgG1 mAb tested in various clinical trials, exhibited similar specificity in cellular binding assays, with an EC_50_ of 0.5 nM (**Table 1**, **Supplementary Figure 1A**). The binding of 22.0405 to ROR1 was also confirmed using human cancer cells that endogenously express ROR1, such as the MCL cell line JeKo-1 (**Supplementary Figure 1B**).

**Table 1:** Antibody binding properties.

Sample	hROR1			mROR1	hROR-2
	*K*_D_ (M)	EC_50_ (M)	Domain	KD (M)	Binding
22.0405	6.0E−8	5.6E−10	Frizzled	2.4E−7	No binding
zilovertamab	9.0E−09	5.0E−10	Ig-like*	–	No binding
R12 scFv	1.4E−09	2.7E−09	N.D.^1^	–	No binding

*aa 39–151 Acro Biosystems, ^1^ junction between Ig-like and Frizzled domain.

In bio-layer interferometry (BLI) molecular binding studies, the apparent affinity of 22.0405 for the full-length extracellular domain of ROR1 was calculated as 60 nM (**Figure 1B**, **Table 1**), which is slightly lower compared to that of zilovertamab-like antibody (9 nM) (**Table 1**, **Supplementary Figure 1C**). The binding epitope of 22.0405 was confirmed to be conformation-dependent as 22.0405 lost its binding capacity to recombinant extracellular ROR1 treated with either reducing agents and/or upon heat inactivation (**Supplementary Figure 1D**). Using recombinant proteins representing the Kringle (KRD), Ig-like (IG), and Frizzled (FRD/CRD) domains of ROR1 (18) as bait in BLI studies, we showed that 22.0405 efficiently captured the Frizzled (FRD/CRD) domain of ROR1 but neither Kringle nor Ig-like domains of ROR1 (**Figure 1C**). This domain has demonstrated to be the most effective domain at ADCC induction during the screening phase. In contrast, the zilovertamab-like antibody recognized only the Ig-like domain of ROR1 (aa 39–151) (**Figure 1D**), consistent with previously reported findings (19).

To demonstrate the ADCC-inducing potential of 22.0405, JeKo-1 cells endogenously expressing ROR1 were incubated with NK cells derived from peripheral blood of healthy donors in the presence of increasing concentrations of 22.0405, zilovertamab-like antibody, or isotype control. Cell killing was assessed after 4 h by flow cytometry. In these settings, 22.0405 induced tumor cell killing with an EC_50_ of approximately 0.08–1 nM depending on the donor, whereas zilovertamab-like antibody could not induce any tumor cell killing (**Figure 1E**). A control antibody, a fully human IgG1 generated from the phage display library against streptavidin, was used as a negative control.

### Afucosylation of 22.0405 increases affinity to CD16 (FcγRIIIA), but its binding to other Fc receptors remains unaffected

To enhance the ADCC-inducing efficacy and potency of 22.0405, by means of increasing its affinity for the FcγRIIIA receptor (CD16) found on NK cells, 22.0405 was produced as an afucosylated IgG1 in CHO-K1 cells using GlymaxX^®^ technology. To confirm that afucosylated 22.0405 (hereinafter called 22.0405.aF) has a higher affinity for the FcγRIIIA receptor compared to its native counterpart, we compared binding of 22.0405.aF with 22.0405 in native form (i.e., containing fucose) to two common allotypes of FcγRIIIA, 158V and 158F (also referred to as 176V and 176F, respectively), using SPR technology. The native form 22.0405 displayed an affinity of 217 and 690 nM for the FcγRIIIA 158V and 158F variants, respectively. 22.0405.aF demonstrated an affinity of 12 and 57 nM for the FcγRIIIA 158V and 158F variants, displaying 17.9- and 12.1-fold increased affinity, respectively (**Table 2**). For comparison, the zilovertamab-like antibody, a native IgG1, displayed an affinity of 570 nM and 3.40 µM for the FcγRIIIA 158V and 158F variants, respectively. These data confirm that 22.0405.aF has a higher affinity for both FcγRIIIA allotypes than 22.0405 in its native (i.e., fucosylated) form, as well as zilovertamab. Afucosylation did not seem to affect 22.0405.aF binding to other Fcγ receptors, including FcγRI (CD64), FcγRIIa (CD32a), and FcγRIIb (CD32b), or to the neonatal Fc receptor (FcRn) (**Supplementary Table 1** and exemplary sensograms, **Supplementary Figure 2**). The afucosylation did not affect the binding or affinity of 22.0405.aF to ROR1.

**Table 2:** Fc binding affinities to FcγRIII variants.

Sample	Affinity *K*_D_ (M)			
	FcyRIIIA (158V)	Fold increased affinity	FcyRIIIA (158F)	Fold increased affinity
22.0405	2.17E−07	18×	6.90E−07	12×
22.0405.aF	1.22E−08		5.70E−08	
zilovertamab	5.51E−07	n.a.	3.40E−06	n.a.

n.a., not applicable.

### Afucosylation of 22.0405 leads to enhanced antibody-dependent cytotoxicity

**Figure 2:**
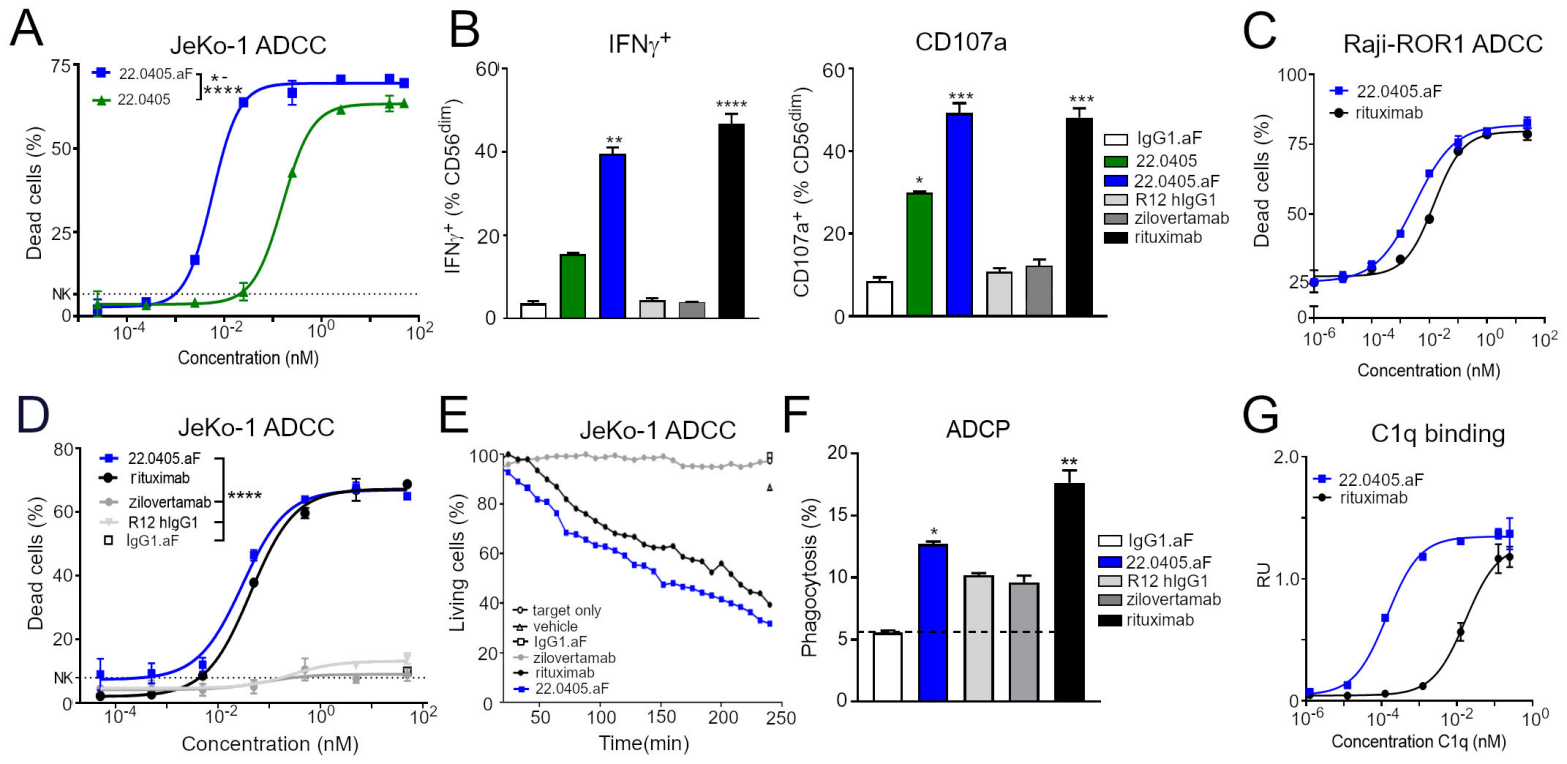
Afucosylation of 22.0405 demonstrates enhanced activation of effector cells and tumor cell killing. **(A)** JeKo-1 target cells were treated with serial dilutions (0.025pM–50 nM) of native or afucosylated formats of 22.0405 and subjected to NK-mediated cellular lysis for 4 h at 37°C with an E:T ratio of 10:1. Cell killing was analyzed by flow cytometry in the presence of live/dead stain. 22.0405.aF was statistically significant from 22.0405 at doses between 0.0025 and 25 nM using two-way ANOVA with Sidak multiple comparison. The graph presents mean ± SD for a representative donor, with experiments performed in duplicates or triplicates for at least three donors. **(B)** PBMCs were incubated with JeKo-1 cells and with either 22.0405, 22.0405.aF, rituximab, zilovertamab, or afucosylated isotype control (IgG1.aF) at 5 nM for 4 h after in which NK cells were analyzed by flow cytometry for IFN-γ or CD107a expression in the presence of Golgi stop. Statistical significance is compared to IgG1.aF using two-way ANOVA with Dunn’s multiple comparison. The graph presents pooled data from four independent donors performed in duplicates, mean ± SD. **(C)** Raji cells transfected with *ROR1* were subjected to NK-mediated cellular lysis for 4 h at 37°C, and cell killing was analyzed by flow cytometry. The data are representative of at least three independent donors tested in duplicates or triplicates, mean ± SD. **(D)** JeKo-1 target cells were treated with serial dilutions of 22.0405.aF, rituximab, zilovertamab, R12 hIgG1, or afucosylated isotype control (IgG1.aF) and NK cells as effector cells for 4 h, followed by analysis of cell killing by flow cytometry. Using two-way ANOVA with Tukey multiple comparison, 22.0405.aF was statistically significant from isotype control (IgG1.aF) R12 IgG1 and zilovertamab from a dose of 0.005 nM and above. The graph is representative of at least three independent donors tested in duplicates or triplicates, mean ± SD. **(E)** JeKo-1 target cells were imaged in the presence of freshly isolated NK cells and either 22.0405.aF, zilovertamab, rituximab, or afucosylated isotype control at 50 nM for cell killing using the Opera Phenix High Content Screening System. Data are representative of three independent donors tested. **(F)** JeKo-1 target cells were treated with either 22.0405.aF, zilovertamab, rituximab, or afucosylated isotype control (IgG1.aF) at 50 nM and subjected for phagocytosis by maturated macrophages analyzed by flow cytometry. Data are representative of three independent donors tested in triplicates, mean ± SD. Statistical analysis was performed using Kruskal–Wallis one-way ANOVA with Dunn’s multiple comparison towards IgG1.aF **(G)** C1q complement protein binding to 22.0405.aF was assessed in ELISA by using MaxiSorp plates coated with 22.0405.aF. Native human C1q complement component was added at different concentrations and C1q polyclonal Ab-HRP was used to detect bound C1q. The data are representative of an experiment from three repetitions performed in duplicates, mean ± SD. *p < 0.05, **p < 0.01, ***p < 0.0001, ****p < 0.0001.

To demonstrate that increased affinity for FcγRIII by afucosylation leads to enhanced ADCC, ROR1-expressing JeKo-1 cells were incubated with NK cells derived from healthy blood donors in the presence of increasing concentrations of either 22.0405 or 22.0405.aF antibody, and cell killing was assessed after 4 h by flow cytometry. 22.0405.aF induced tumor cell killing with an EC_50_ of approximately 6–30 pM compared to an EC_50_ of 160–900 pM for native 22.0405 with the tested donors (**Figure 2A**, **Supplementary Figure 3A**). In experiments similar to ADCC, using healthy donor-derived PBMCs as effector cells and JeKo-1 as target cells, 22.0405.aF increased the percentage of NK cells expressing IFN-γ (**Figure 2B**, left) and TNF-α (**Supplementary Figure 3B**, left). IFN-γ-positive cells increased more than twofold compared to 22.0405 and eightfold compared to the afucosylated isotype control (IgG1.aF). TNF-α was increased by 22.0405.aF treatment by sixfold compared to the afucosylated isotype control. Similarly, 22.0405.aF triggered a fivefold increase in the number of NK cells expressing the degranulation marker CD107a on the cell surface, compared to isotype control IgG1.aF and almost twofold increase compared to 22.0405 (**Figure 2B**, right). Thus, it appears that 22.0405.aF increases the number of NK cells engaged in ADCC, compared to 22.0405, leading to more efficient target cell killing. In addition, 22.0405.aF increased Fas ligand (FasL) expression on NK cells (**Supplementary Figure 3B**, right), which has been previously shown to induce extrinsic apoptosis of target cells (20). The activation of NK cells by 22.0405.aF was in a range similar to that observed for rituximab.

To assess how the different levels of ROR1 on target cells affect 22.0405.aF-mediated ADCC, NK-based cytotoxicity assays were performed using tumor cell lines expressing different levels of ROR1. First, the capability of 22.0405.aF to induce NK cell-mediated ADCC towards Raji cells overexpressing human ROR1 (Raji-ROR1, ~25K ROR1/cell) was assessed using flow cytometry. In the assay, 22.0405.aF induced a potent concentration-dependent ADCC towards Raji-ROR1 cells, with an average EC_50_ of approximately 2 pM, depending on the donor (**Figure 2C**). The ADCC effect observed was similar to the ADCC induced by the rituximab antibody. To ensure that the cell killing was ROR1-dependent, the ADCC induction capability was similarly assessed against wild-type Raji cells. 22.0405.aF did not induce statistically significant ADCC towards wild-type Raji cells, expressing very low levels of ROR1 (~500–1,300 ROR1/cell), indicating specificity and threshold levels required for ADCC induction (**Supplementary Figure 3C**).

To evaluate the ability of 22.0405.aF to induce NK cell-mediated ADCC against tumor cells that naturally express ROR1, NK cytotoxicity assays were performed using JeKo-1 cells as targets (5–10K ROR1/cell). The ADCC efficacy of 22.0405.aF was also compared to that of the anti-ROR1 monoclonal antibodies zilovertamab and R12, as well as rituximab. 22.0405.aF induced potent concentration-dependent ADCC towards JeKo-1 cells with an EC_50_ of approximately 6–80 pM depending on the donor (**Figure 2D**). Neither zilovertamab- nor R12-like antibody was able to induce any significant tumor cell killing against JeKo-1 cells, nor the afucosylated isotope control antibody. The level of ADCC induction by 22.0405.aF was comparable to rituximab, but in contrast to rituximab, 22.0405.aF did not induce killing of naïve B cells (**Supplementary Figure 3D**). The fast time-dependent NK cell engagement and tumor cell killing by 22.0405.aF within 60 min and 4 h was confirmed using the Opera Phenix High Content Screening System (**Figure 2E**, **Supplementary Video 1**). Lastly, potent tumor cell killing with an EC_50_ of 20 pM (*n* = 6 donors), similar to rituximab, was confirmed using total PBMCs as effector cells (**Supplementary Figure 3E**).

### 22.0405.aF demonstrates other Fc-mediated effector functions

We hypothesized that 22.0405.aF may induce Fc-mediated effector functions, other than ADCC, such as antibody-dependent cell-mediated phagocytosis (ADCP) and complement-dependent cytotoxicity (CDC). The ability of 22.0405.aF to induce ADCP was assessed through measuring the capacity of macrophages, matured from healthy donor PBMCs, to engulf fluorescently labeled JeKo-1 target cells. The killing effect of macrophages was analyzed by flow cytometry. In the presence of 50 nM 22.0405.aF, macrophages phagocytosed approximately two times as many JeKo-1 cells when compared to phagocytosis in the presence of afucosylated isotype control antibody (IgG1.aF), which was in similar range to ADCP induced by rituximab in the same experiment. Zilovertamab- and R12-like antibodies induced a slight, but not statistically significant, increase of ADCP compared to isotype control in the experiments (**Figure 2F**).

To investigate whether 22.0405.aF can induce complement activation, the capacity of 22.0405.aF to bind C1q, the first protein in the complement cascade, was assessed in an ELISA-based assay. To this end, different concentrations of native human C1q complement protein were added to 22.0405.aF immobilized on MaxiSorp plates. After binding and washing, HRP-conjugated anti-C1q antibodies were used to detect 22.0405.aF-bound C1q. 22.0405.aF bound C1q specifically and in a concentration-dependent manner, indicating that afucosylation did not abolish the Cq1 binding and that 22.0405.aF may have an intrinsic capacity to induce CDC (**Figure 2G**).

### 22.0405.aF induces potent ADCC against primary chronic lymphocytic leukemia cancer cells

**Figure 3:**
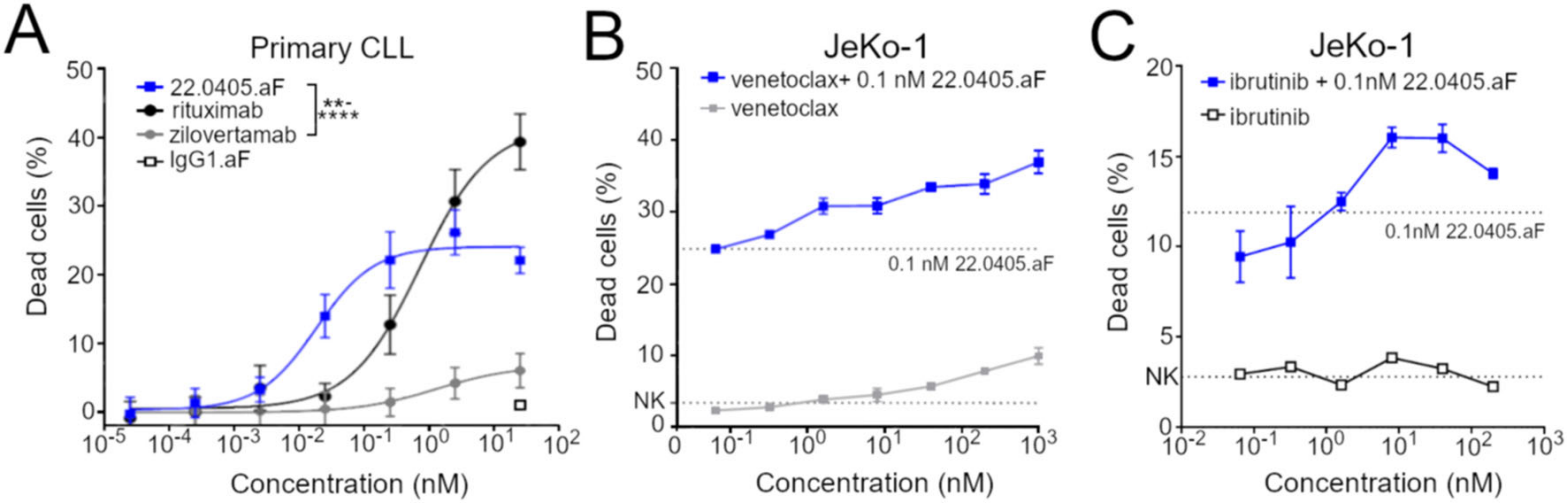
22.0405.aF demonstrates potent activity again primary CLL patient material and good combinatorial activity with ibrunitib and venetoclax. **(A)** PBMCs from patients with B-CLL, containing 60%–95% malignant B lymphocytic cells, were co-cultured with healthy donor-derived NK cells in the presence or absence of various concentrations of 22.0405.aF, zilovertamab-like antibody, or rituximab for 4 h, and tumor cell killing was analyzed by flow cytometry. The data are representative of pooled data from three patients, performed in duplicates or triplicates. The data are presented as mean ± SD. 22.0405.aF is statistically significant compared to zilovertamab from the dose of 0.025 nM using two-way ANOVA with Tukey multiple comparison. **(B, C)** JeKo-1 cells were incubated at various concentrations of ibrutinib or venetoclax followed by 0.1 nM of 22.0405.aF in the presence of freshly isolated NK cells. Cell killing was analyzed by flow cytometry. The data are presented as mean ± SD. The dotted line represents 22.0405.aF’s effect alone. Graphs are representative examples from at least three donors tested in duplicates or triplicates. **p < 0.01, ****p < 0.0001.

Immunotherapies targeting CD20, antibodies such as rituximab and obinutuzumab, are often integrated into first-line B-CLL treatment in the Western world (13). Although efficient, these therapies induce not only ADCC towards malignant, but also healthy B cells, thereby limiting its application. ROR1 is expressed primarily on malignant B cells; thus, 22.0405.aF may be particularly suitable for treatment of B-cell malignancies. To demonstrate that 22.0405.aF can kill primary B-CLL cells, PBMCs from patients with B-CLL (*n* = 3), containing 65%–99% ROR1-positive malignant B-lymphocytic cells, were co-cultured with healthy donor-derived NK cells in the presence or absence of various concentrations of 22.0405.aF, zilovertamab-like antibody, or rituximab. 22.0405.aF displayed significant ADCC against primary B-CLL cells, with an average EC_50_ of 18 pM that was approximately fourfold lower than EC_50_ of rituximab (**Figure 3A**). Importantly, 22.0405.aF did not induce ADCC towards isolated non-malignant B cells from healthy donors, unlike rituximab that is known to deplete healthy B cells (**Supplementary Figure 2E**). In contrast, a zilovertamab-like antibody demonstrated only a minor killing effect. The data confirm the potential of 22.0405.aF as an immunotherapy for the treatment of B-CLL.

### 22.0405.aF demonstrates additive tumor cell killing with standard-of-care therapies

Oral treatments for B-CLL, such as the BTK inhibitor ibrutinib and the BCL-2 inhibitor venetoclax, often in combination with the ADCC-inducing therapies obinutuzumab or rituximab, have demonstrated good clinical responses in first-line treatment of patients with B-CLL. Yet, a portion of patients are intolerant to these treatments, and most patients develop treatment resistance over time (21). To investigate the tumor cell-killing potential of 22.0405.aF in combination with ibrutinib or venetoclax, JeKo-1 cells were incubated with various concentrations of either ibrutinib or venetoclax overnight. Subsequently, NK cells and a fixed concentration of 22.0405.aF at 0.1 nM were added to target cells and ADCC was assessed using flow cytometry after 4 h of incubation. 22.0405.aF demonstrated an additive effect in cell killing in the presence of venetoclax or ibrutinib (**Figures 3B, C**), thus supporting that the addition of 22.0405.aF to current treatment paradigms may yield stronger clinical responses in patients with cancer treated with BTK inhibitors or venetoclax.

### 22.0405.aF induces anti-tumor efficacy *in vivo* against leukemic tumor cells

**Figure 4:**
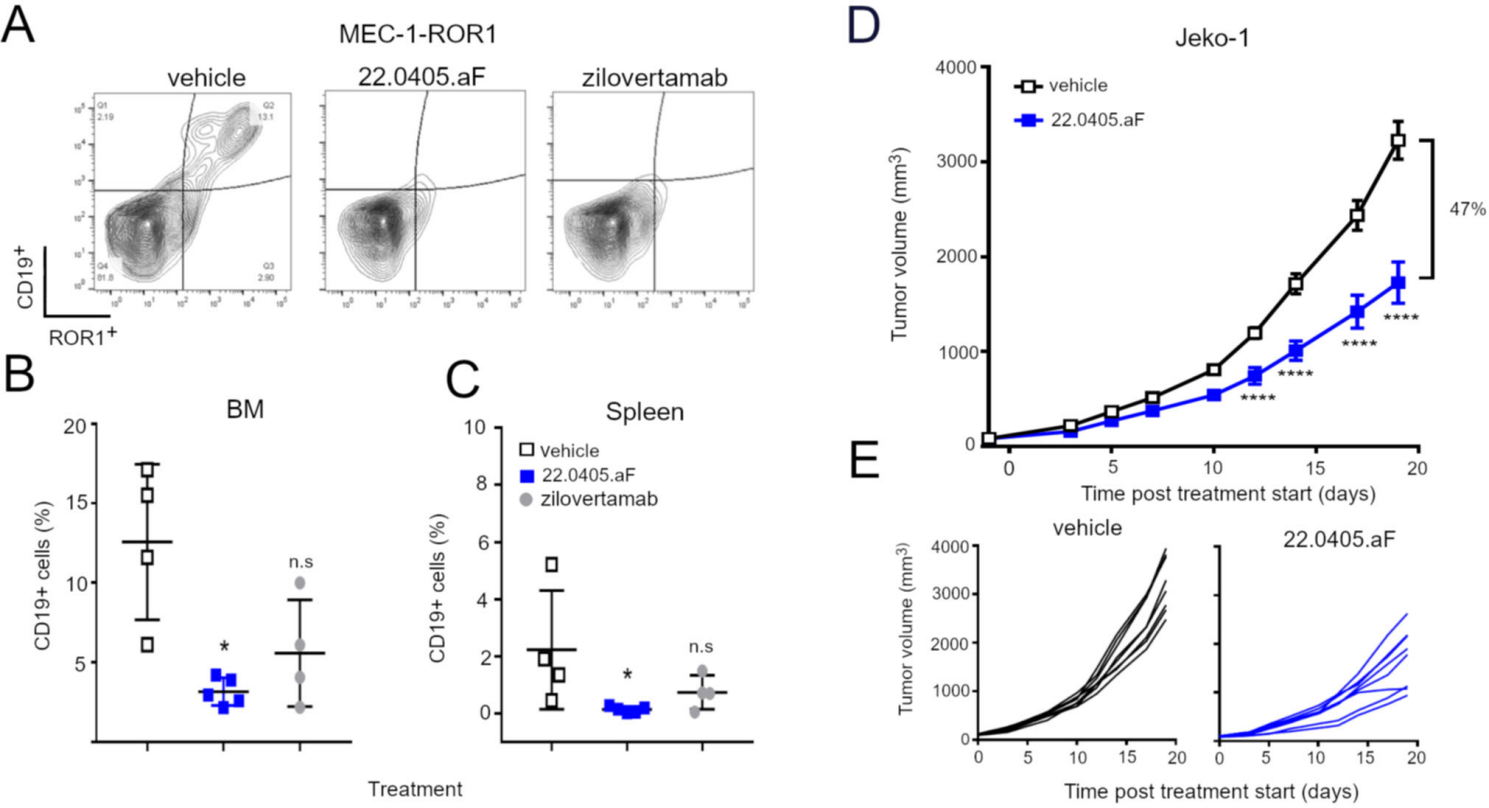
22.0405.aF demonstrates anti-tumor efficacy against disseminated B-CLL model. **(A–C)** SCID mice were inoculated IV with MEC-1-ROR1 cells. After 1 week, mice received intravenous treatments of 22.0405.aF or zilovertamab at 10 mg/kg twice a week for a total of six doses. Four weeks after inoculation, the mice were terminated, and the bone marrow and spleen were analyzed for CD19 and ROR1-positive B-CLL cells. The graph represents the mean ± SD, *n* = 4–5. Statistical significance was considered using one-way analysis of variance (ANOVA), with Kruskal–Wallis multiple comparison, *p < 0.05. **(D)** CD34^+^ humanized NCG-hIL15 mice were inoculated subcutaneously with JeKo-1 cells and were treated with six doses of 22.0405.aF at 10 mg/kg twice weekly starting when the tumor volume was approximately 82 mm^3^. The graph represents the mean ± SEM, *n* = 8. The individual tumor growth is shown in **(E)**. Statistical significance was considered using two-way ANOVA, with Bonferroni multiple comparison. *p < 0.05, ****p < 0.0001.

To investigate the potential of 22.0405.aF to control B-CLL *in vivo*, CB-17 severe immunodeficient (SCID) mice were injected intravenously with MEC-1-ROR1 cells, a human CLL cell line overexpressing human ROR1 (~28–32K ROR1/cell). A week after tumor cell inoculation, the mice received intravenous treatments of vehicle, 22.0405.aF, or zilovertamab-like antibody, at 10 mg/kg, twice a week for a total of six doses. Four weeks after inoculation, the mice were terminated, and the bone marrow and spleen were analyzed for the presence of CD19- and ROR1-positive CLL cells. In this model, 22.0405.aF demonstrated a statistically significant reduction in the numbers of MEC-1-ROR1 cells in both the bone marrow and the spleen (**Figures 4A–C**). Zilovertamab-like antibody demonstrated lower anti-tumor efficacy compared to 22.0405.aF, which was not statistically significant.

### 22.0405.aF activates NK cells in the tumor environment and induces anti-tumor efficacy

The anti-tumor efficacy of 22.0405.aF was assessed in human CD34^+^ hematopoietic stem cell (huHSC) humanized NCG-hIL15 mice inoculated subcutaneously with JeKo-1 cells on the right flank/shoulder. When the average tumor volume reached approximately 82 mm^3^, mice were grouped and randomized according to huHSC donor, humanization level, and tumor volume. Subsequently, mice were treated with intravenous injections of vehicle or 22.0405.aF, at 10 mg/kg body weight, biweekly for six doses. 22.0405.aF demonstrated statistically significant tumor growth inhibition with 47% decrease in tumor volume compared to the vehicle treatment group upon termination (**Figure 4D**). The individual tumor growth curves are shown in (**Figure 4E**). For comparison, rituximab in combination with CHOP inhibited JeKo-1 tumor growth in NSG mice with approximately 60% (22).

**Figure 5:**
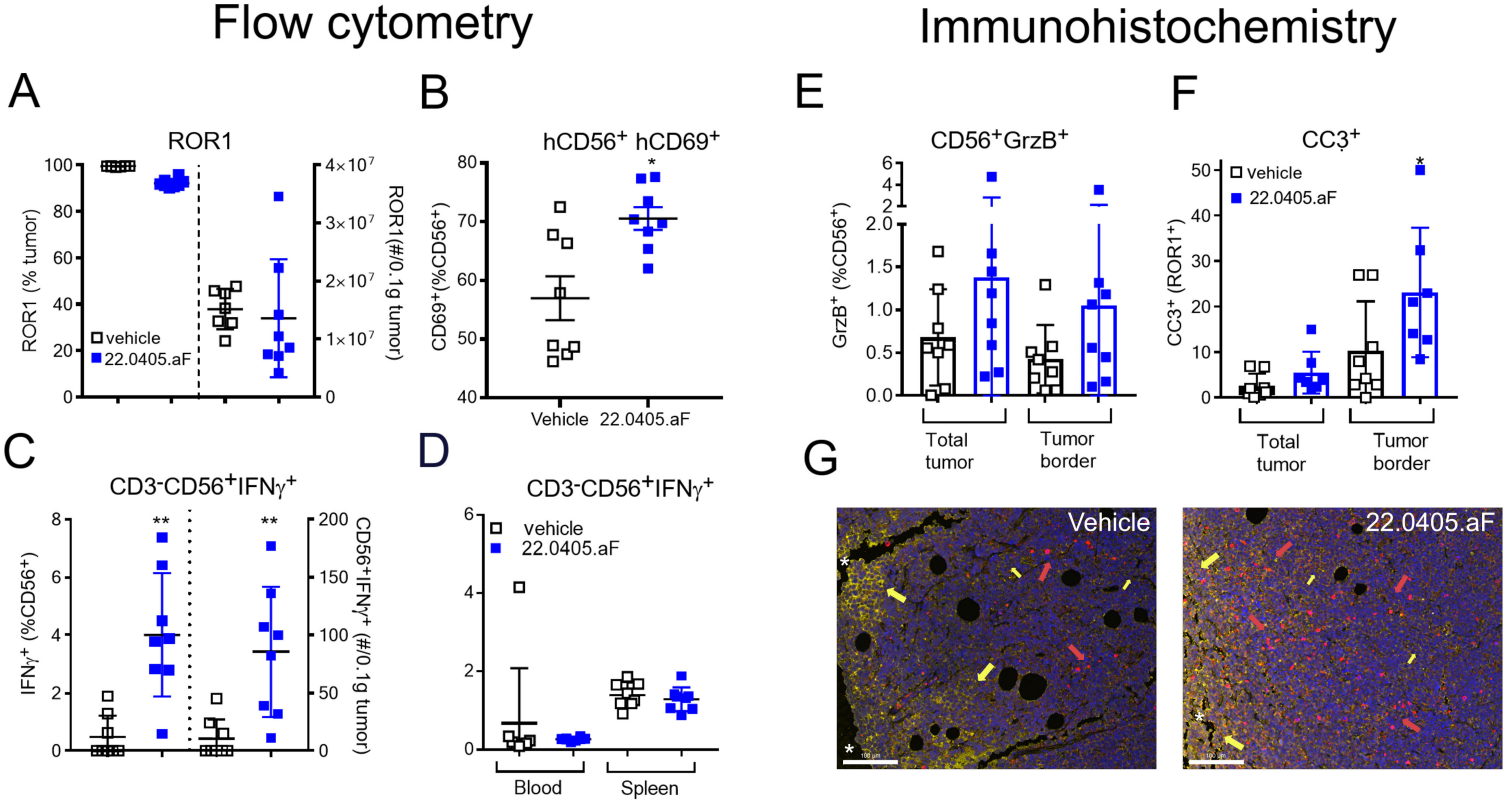
22.0405.aF activates effector cells locally, leading to anti-tumor efficacy. CD34^+^ HSC humanized NCG-hIL15 mice (*n* = 8/group) were inoculated subcutaneously with JeKo-1 mice and were treated either with vehicle or with six doses of 22.0405.aF at 10 mg/kg twice weekly starting when the tumor volume was, on average, 82 mm^3^. At termination of the experiment, tumors and spleens were removed, cut in half, and processed together with terminal bleeding for FACS analysis. **(A)** ROR1 expression on tumor cells was analyzed by flow cytometry. **(B)** NK cell activation in the form of CD69 upregulation by 22.0405.aF treatment was analyzed by flow cytometry. **(C)** IFN-γ-positive NK cells in tumors analyzed by flow cytometry, percentage, and number of cells/0.1 µg tumor. **(D)** Lack of IFN-γ upregulation in blood and spleen NK cells analyzed by flow cytometry. **(E–G)** The second halves of the same tumors as described above were PFA fixated and processed for fluorescent immunohistochemistry. **(E)** Granzyme B expression in tumor NK cells. **(F)** Cleaved caspase-3 expression in ROR1-positive tumor cells. **(G)** Representative IHC analysis of vehicle- and the respective 22.0405.aF-treated tumors. The line represents 100 µm. Yellow arrows indicate ROR1 expression in yellow, and red arrows denote cleaved caspase-3 in red. The white stars indicate the tumor edge where the ROR1 expression is strongest, while the rest of the tumor has a weaker ROR1 expression. The data are presented as mean ± SD, *n* = 7–8. Statistical significance was considered using Kruskal–Wallis, non-parametric ANOVA, *p < 0.05, **p < 0.01 for multiple comparisons or Mann–Whitney non-parametric comparison for single comparison. In panel F, *p* presents one-tailed significance; otherwise, *p* presents two-tailed significance.

To confirm the therapeutic mode of action of 22.0405.aF *in vivo*, the tumors, spleens, and blood were collected at termination of the experiment and processed to single cells, stained with biomarkers for different effector cell populations and status of cell activation, and analyzed by flow cytometry. ROR1 expression was shown to be retained on 90%–95% of tumor cells after 22.0405.aF treatment, indicating that 22.0405.aF exposure does not significantly downregulate the percentage of ROR1-positive cells in these settings (**Figure 5A**). 22.0405.aF treatment induced statistically significant activation of tumor-resident NK cells, measured as an increased percentage of NK cells expressing CD69 (**Figure 5B**) or IFN-γ compared to vehicle-treated tumors. The intracellular IFN-γ levels in CD56^+^ NK cells in the tumor environment was increased fivefold upon 22.0405.aF administration compared to vehicle control (**Figure 5C**), which was observed both as the number of positive cells and as a percentage of positive CD56^+^ NK cells. These data are consistent with the *in vitro* data where activation of NK cells demonstrated potent IFN-γ production and tumor killing towards ROR1-expressing cells. The induction of IFN-γ was tumor specific as no increase of IFN-γ levels was seen in either blood or spleen NK cells, demonstrating that 22.0405.aF activates NK cells to release IFN-γ only in the tumor environment and not systemically (**Figure 5D**). These data demonstrate the target specificity and safety of 22.0405.aF in the form of NK cell activation only in the presence of ROR1.

In addition to flow cytometry, paraffin-embedded JeKo-1 tumors, removed at termination, were analyzed for ROR1 target expression, immune activation, and cell death. The immunohistochemical analysis showed that 22.0405.aF does not downregulate ROR1 antigen expression significantly on tumor cells, as no significant differences in either percentage of positive cells or obvious change in intensity of the ROR1 staining were observed, between vehicle-treated tumors and 22.0405.aF-treated tumors, confirming the results from flow cytometry analysis. However, one should note that IHC does not discriminate cytosolic versus cell surface expression of ROR1. Slightly increased numbers of granzyme B-positive cells were found in tumors of 22.0405.aF-treated mice compared to vehicle-treated mice, albeit this was not statistically significant (**Figure 5E**). A statistically significant increase of cleaved caspase-3-positive cells and an approximately twofold increase of double-positive cells for ROR1 and caspase-3 (one-tailed significance) were observed by 22.0405.aF treatment compared to vehicle-treated tumors, indicating that, together with IFN-γ upregulation by FACS analysis, 22.0405.aF activates NK cells *in vivo* and induced tumor cell killing of ROR1-positive cancer cells (**Figures 5F, G**).

## Discussion

The encouraging clinical outcomes observed with zilovertamab and zilovertamab-vedotin have reinforced ROR1 as a promising therapeutic target in both hematological malignancies and solid tumors. While ROR1 is predominantly expressed during embryonic and fetal development, its expression is largely absent in mature tissues, with only minimal levels detected in adipose tissue, and to a lesser extent in the pancreas, lung, thyroid, stomach, and a subset of intermediate B cells (23–25). However, in the context of malignancy, aberrant ROR1 expression has been widely documented across various cancers, supporting its role in tumorigenesis and drug resistance (26, 27).

ROR1 overexpression was first identified in B-cell CLL, where it is expressed on the surface of malignant cells in nearly all patients (94%) according to flow cytometry analysis (28). Moreover, ROR1 expression increases as CLL progresses, suggesting its potential as a prognostic biomarker. Beyond CLL, ROR1 has been implicated in other hematologic malignancies, including acute lymphoblastic leukemia (ALL), non-Hodgkin lymphomas (NHLs), and myeloid malignancies (27–30). Additionally, ROR1 expression is prevalent in solid tumors such as ovarian cancer, breast cancer, particularly triple-negative breast cancer (TNBC), and lung cancer (26, 31–33).

Given its restricted expression in normal adult tissues and its overexpression in malignancies, ROR1 represents an ideal therapeutic target. Several ROR1-targeting modalities, including monoclonal antibodies (e.g., zilovertamab), antibody–drug conjugates (e.g., zilovertamab-vedotin), and chimeric antigen receptor T-cell (CAR-T) therapies, have demonstrated potent anti-tumor efficacy in preclinical and clinical studies. The observed adverse effects appear to be largely dependent on the treatment modality, with CAR-T cell therapy-associated cytokine release syndrome and ADC-associated neurotoxicity being among the most frequently reported toxicities (26, 27).

To further expand the therapeutic potential of ROR1-targeting strategies, we developed a novel monoclonal antibody, 22.0405.aF, which was engineered for enhanced ADCC. In contrast to zilovertamab, 22.0405.aF recognizes a distinct epitope on ROR1 and demonstrates markedly superior ADCC activity. Afucosylation of 22.0405.aF increased its affinity for the FcγRIII receptor on NK cells relative to the native form, thereby promoting more robust NK cell activation and enhanced ADCC induction. *In vitro*, 22.0405.aF induced a ~30-fold increase in ADCC potency against JeKo-1 cells compared with its native counterpart. Despite the limited ROR1 surface density on these cells, approximately 20-fold lower than the antigen target levels for rituximab, the ADCC activity of 22.0405.aF was comparable to that of rituximab. Notably, 22.0405.aF also displayed greater cytotoxic potency against primary samples from patients with CLL relative to rituximab. Importantly, unlike rituximab, 22.0405.aF did not mediate cytotoxicity toward naïve B cells, suggesting a potentially improved safety profile in hematologic malignancies when compared to CD20-targeting therapies. Beyond hematologic cancers, 22.0405.aF also demonstrated potent activity against ROR1-positive solid tumors, highlighting its translational potential for TNBC, ovarian cancer, and other ROR1-expressing malignancies.

Despite significant advances in the treatment of hematologic cancers with BTK inhibitors (e.g., ibrutinib) and BCL-2 inhibitors (e.g., venetoclax), resistance and disease relapse remain major challenges (21). Notably, high ROR1 expression and ROR1-mediated signaling have been associated with resistance to both ibrutinib and venetoclax in CLL (34). In MCL, ibrutinib-resistant tumors exhibit aberrant ROR1 expression, which interacts with CD19 to form a functional signaling complex, promoting cell survival independent of the BCR–BTK axis (35). Furthermore, studies have shown that destabilization of ROR1 enhances ibrutinib sensitivity in CLL *in vivo* (36). Clinical trials evaluating the combination of BTK or BCL-2 inhibitors with ROR1-targeting therapies have yielded encouraging results, further underscoring the critical role of ROR1 in cancer progression and therapy resistance. Consistent with these findings, our *in vitro* ADCC assays demonstrated that combining 22.0405.aF with either ibrutinib or venetoclax enhanced tumor cell killing, supporting the rationale for future combination therapy approaches integrating 22.0405.aF with BTK or BCL-2 inhibitors.

22.0405.aF induced significant tumor growth inhibition in both MEC-1-ROR1 and JeKo-1 xenografts, accompanied by robust activation of tumor-infiltrating NK cells in humanized mouse models. Notably, 22.0405.aF increased both the frequency and functional activation of CD56^+^ NK cells within the tumor microenvironment, as reflected by elevated CD69 expression and a fivefold increase in intracellular IFN-γ production. Importantly, this increase of IFN-γ was spatially restricted to the tumor site, as no systemic increase of IFN-γ by NK cells was detected in blood or spleen, underscoring the tumor-specific immune engagement and supporting a favorable safety profile. Beyond NK activation, the antibody promoted caspase-3-dependent apoptosis of ROR1^+^ tumor cells without inducing significant, irreversible antigen downregulation, suggesting a reduced risk of resistance through target loss, a common barrier observed in many therapies. Although NK cell activation in the form of IFN-γ was seen in Jeko-1 models, we cannot totally exclude the potential cell killing by other effector cells such as macrophages and T cells. However, in this model, macrophages were sparse due to the model that supports NK cell development rather than the development of myeloid compartment. Furthermore, the residual macrophages were observed rather in the middle of the tumor, whereas a higher cell death was seen near the rim area, where also the ROR1 expression was strongest, in accordance with ROR1 being involved in cell migration and invasion (37).

In conclusion, our preclinical studies have demonstrated that 22.0405.aF exhibits superior ADCC compared to zilovertamab, a native ROR1 antibody advanced to Phase 3 clinical trials. The afucosylation of 22.045.aF significantly enhances its affinity for FcγRIII receptors on NK cells, leading to robust NK cell activation and tumor-specific cytotoxicity. Importantly, 22.0405.aF selectively targets ROR1-positive malignant cells without affecting healthy B cells, suggesting a favorable safety profile compared to current CD20-targeting therapies. *In vivo* studies further validated the therapeutic potential of 22.0405.aF, showing the significant tumor growth inhibition and activation of tumor-resident NK cells. Additionally, 22.0405.aF demonstrated additive tumor cell-killing effects when combined with standard-of-care therapies such as ibrutinib and venetoclax, highlighting its potential for combination treatment strategies. Although there are various ROR1-specific antibodies currently in clinical development, to the best of our knowledge, 22.0405.aF is the first fully human anti-ROR1 monoclonal antibody in clinical development specifically designed and engineered for optimal ADCC induction. Our preclinical *in vitro* and *in vivo* data strongly support the therapeutic potential of 22.0405.aF for both hematologic malignancies and solid tumors. 22.0405.aF, now named PBA-0405, has recently successfully completed a Phase 0 clinical trial (NCT06273852) as an early proof of concept in ROR1-positive patients with head and neck squamous cell carcinomas and soft tissue sarcomas. Simultaneously, preparations are underway for Phase 1 clinical trials focusing first on hematologic malignancies, particularly CLL and MCL as proof of concept, thereafter pursuing solid tumor indications such as TNBC.

## Data Availability

The original contributions presented in the study are included in the article/**Supplementary Material**. Further inquiries can be directed to the corresponding authors.
