# Supplementary material for: A novel fully human anti-ROR1 antibody PBA-0405 optimized for ADCC, induces potent anti-tumor activity against both solid and hematological malignancies
Source: Front Immunol. 2025 Dec 17;16:1711509. doi: 10.3389/fimmu.2025.1711509 (PMC12753870; doi:10.3389/fimmu.2025.1711509)
Supplement: Supplementary file 1 [file DataSheet1.docx]

Supplementary Material

# Supplemental data

# Supplementary Figures and Tables

## Supplementary Figures

##
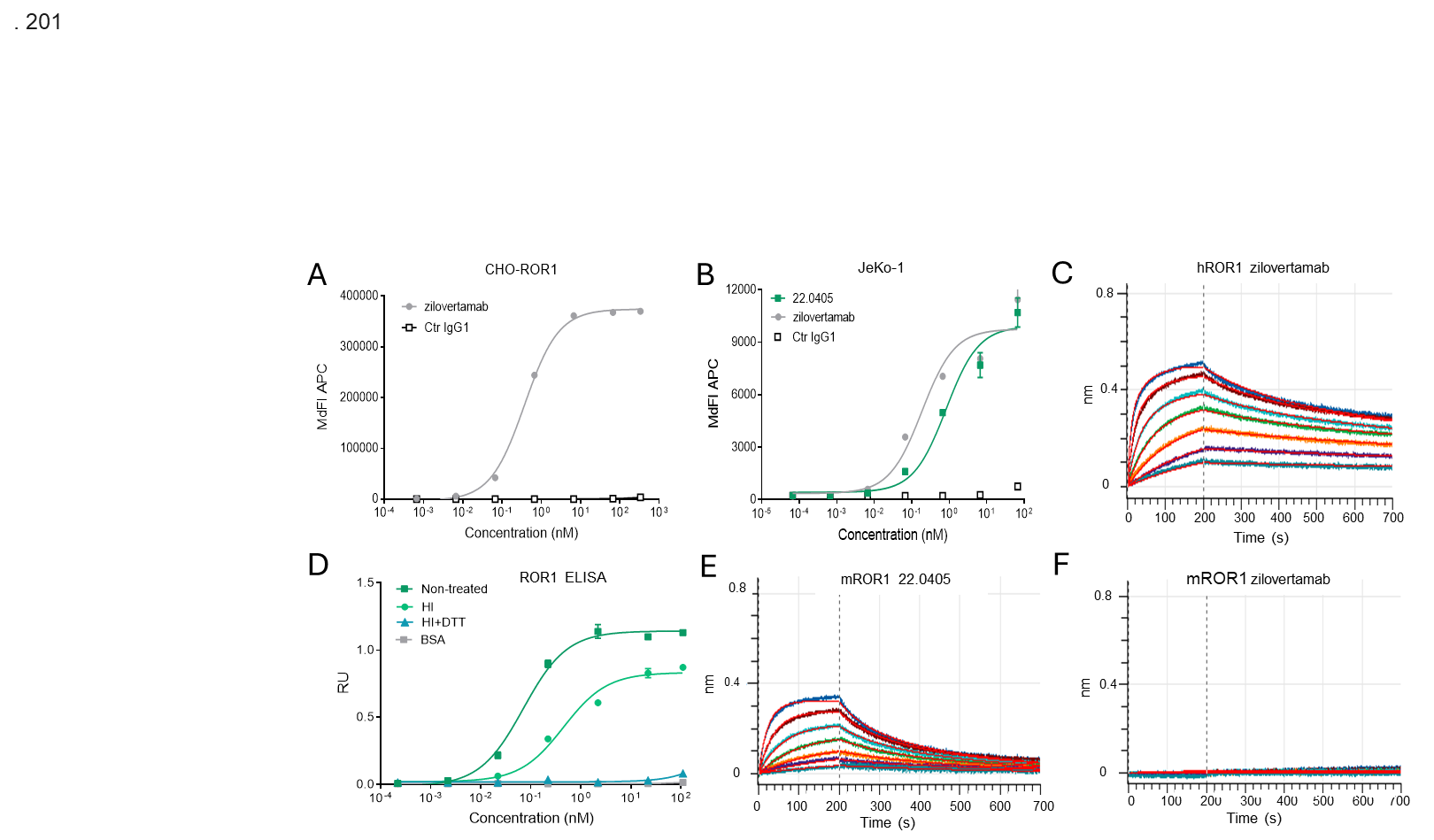


**Supplementary Figure 1.**  **Binding properties of 22.0405 and zilovertamab-like antibody**

(A) Different concentrations of zilovertamab-like antibody were titrated on CHO overexpressing ROR1, ROR2 or empty vector (EV) control cell line and the binding of zilovertamab-like antibody was analyzed by flow cytometry. (B) Binding of native 22.0405 and zilovertamab-like antibody to JeKo-1 cell line analyzed by flow cytometry. (C) BLI binding analysis of zilovertamab-like antibody to recombinant extracellular ROR1 coated chips at doses between 3.31-200 nM. (D) 22.0405 binding to DTT and/or heat-inactivated recombinant ROR1 assed by ELISA. (E) 22.0405 or (F) zilovertamab-like antibody binding to murine recombinant ROR1 assessed at different concentration by BLI binding assay. All graphs are an exemplary experiment of minimally three experiments performed in duplicates or triplicates. The cellular binding experiment graphs show the mean ± standard deviation (SD).


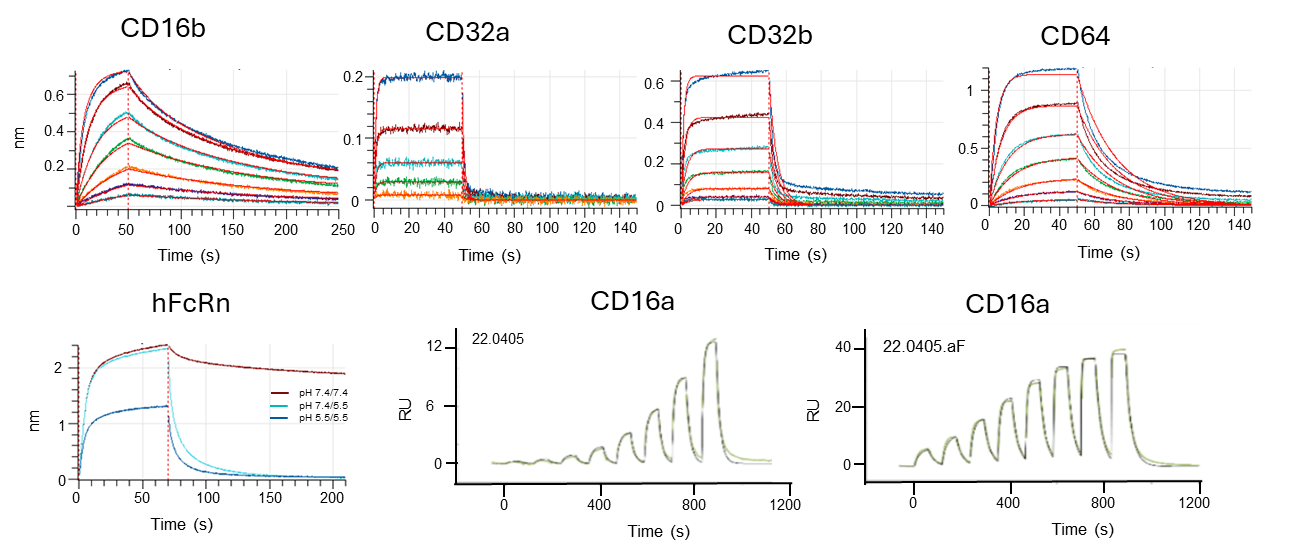


**Supplementary Figure 2.** **FcγR binding properties, exemplary sensograms**

Binding properties of 22.0405.aF to recombinant FcγR receptors was tested in BLI or SPR at doses between 3-200 nM for CD16b, 240-4000 nM for CD32a, 31-2000 nM for CD32b, and 8-500 nM for CD64. Red line indicates fitting. Binding to recombinant hFcRn captured as a ligand on the surface of SAX2 biosensor was performed with different pH, 7.4/7.4, 7.4/5.5 or 5.5/5.5 and measured on Octet RED384 instrument. Binding of 22.0405 and 22.0405.aF was compared in SPR against recombinant CD16a captured as ligand on the surface of SA chip at doses between 7.8-1000 nM. Black lines indicates fitting. All graphs are an exemplary experiment of minimally three experiments performed. Binding affinities measured are indicated in Table 2 and supplementary Table1.


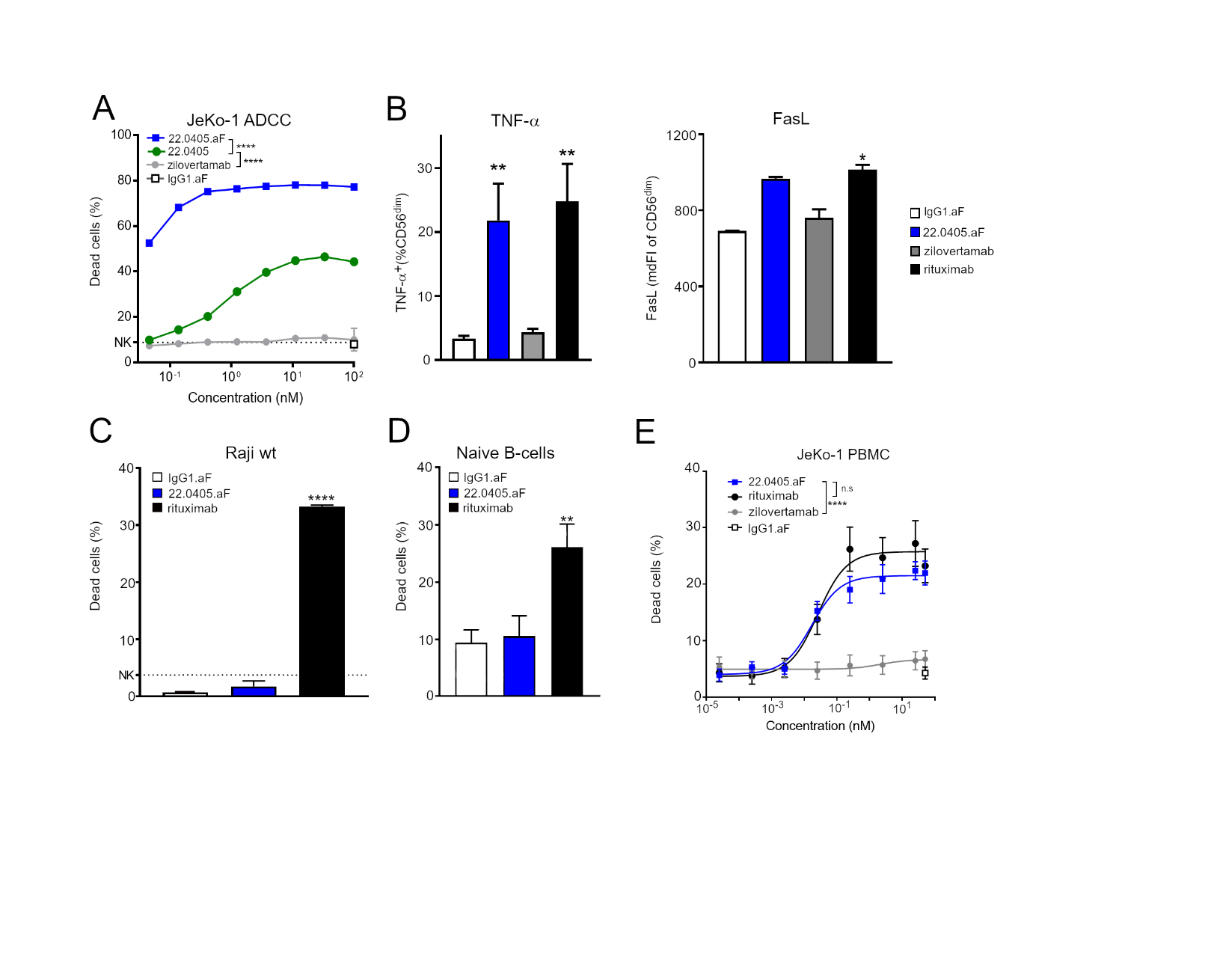


**Supplement Figure 3. Afucosylation of 22.0405 demonstrates potent activation of effector cells and tumor cell killing** (A) JeKo-1 target cells were treated with serial dilutions (0.025pM-50 nM) of native- and afucosylated formats of 22.0405 and subjected to NK-mediated cellular lysis for 4 h at 37°C. Cell killing was analyzed by flow cytometry in the presence of live/dead stain. The graph is a presentation of the donors with highest differences between the different 22.0405 formats. The difference between 22.0405.aF and 22.0405 tumor cell killing is statistically significant in all doses compared to 22.0405 and 22.0405 tumor cell killing is statistically significant compared to zilovertamab-like antibody starting from 0.14 nM (2-way ANOVA with multiple comparisons). (B) Healthy donor derived PBMCs were incubated with JeKo-1 cells and either with 22.0405.aF, rituximab, zilovertamab or isotype control at 5 nM for 4 hours after which CD56dim NK cells were analyzed for TNF-α. (C) Samples in B were analyzed for FASL expression by flow cytometry. Statistical differences are compared to IgG.aF. (D) Raji wt cell or (E) naïve B cells in presence of JeKo-1 cells were subjected to NK-mediated cellular lysis for 4 h at 37°C. The cell killing was analyzed by flow cytometry. (F) JeKo-1 target cells subjected to total PBMC ADCC for 4 h at 37 ºC cells analyzed by flow cytometry. 22.0405.aF is statistically significant from zilovertamab from concentration of 0.25 nM using 2-way ANOVA with Tukey’s multiple comparison. All the NK experiment result shows one representative donor (at least out of 3 tested donors), tested in duplicates, or pooled donors, values are mean and SD. PBMC ADCC shows pooled data from 6 different donors. *p< 0.05 , **p< 0.01, ****p< 0.0001


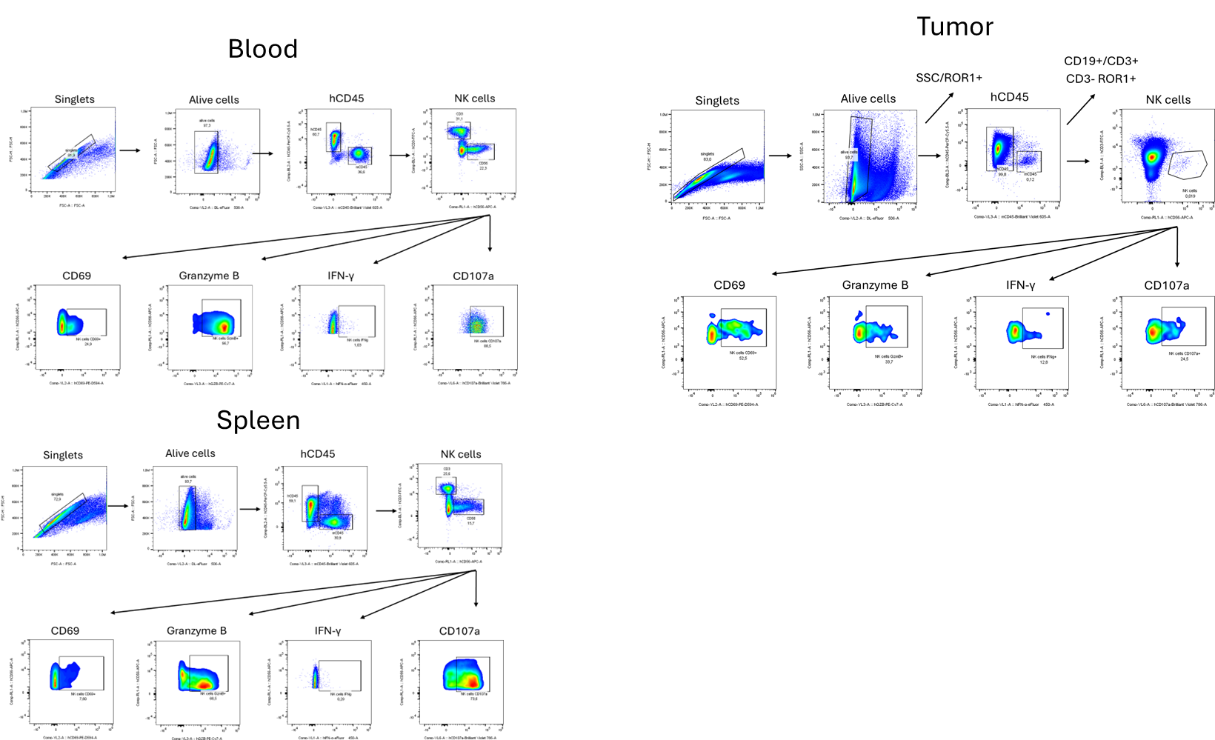


Suppelementary Figure 4. Flow cytometry gating strategy. JeKo-1 tumors, spleens and blood from huHSC-NCG-hIL15 JeKo-1 experiments (n= 8 / group) were processed and stained according to materials and methods. The graphs above are exemplary gating strategies for blood, spleens and tumors, respectively.

## Supplementary Tables

**Supplementary Table1, FcR affinities**

|  | **Affinity K_D_ (M)** | | | | |
| --- | --- | --- | --- | --- | --- |
| **Sample** | **FcyRI** | **FcyRIIA 167R*** | **FcyRIIA 167H*** | **FcyRII B/C** | **FcyRIIIB*** |
| **22.0405.aF** | (1.01 ± 0.07) × 10^-10^ | (1.2 ± 0.2) × 10^-6^ | (8.9 ± 0.9) × 10^-7^ | (4.1 ± 0.6) × 10^-6^ | (3.5 ± 0.9) × 10^-7^ |
| **zilovertamab** | (5.4 ± 0.5) × 10^-11^ | (3.2 ± 0.6) × 10^-6^ | (2.2 ± 0.9) × 10^-6^ | > 4 × 10^-6^ | > 4 × 10^-6^ |
| * Steady state affinity | |  |  |  |  |

**Supplementary Table 2, JeKo-1 tumor weights**

| **Group** | **Treatment** | **TW (g)** | **TW SEM** | **No** |
| --- | --- | --- | --- | --- |
| 1 | **vehicle** | 3.46 | 0.148 | 8 |
| **2** | **22.0405.aF** | 2.30 | 0.213 | 8 |
| TW= Tumor weight | |  |  |  |

## Supplementary Video

**Supplementary Video 1**

**PB004.405.af**

**Rituximab**


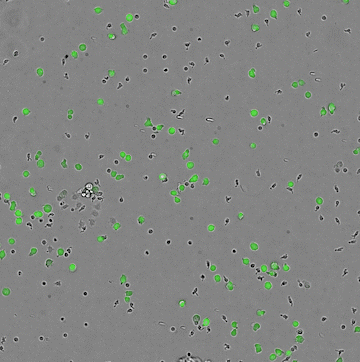

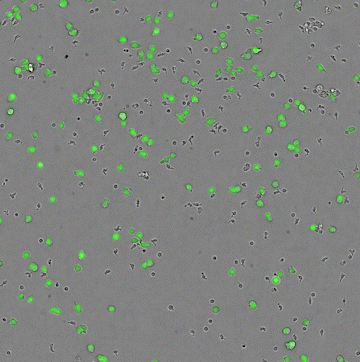

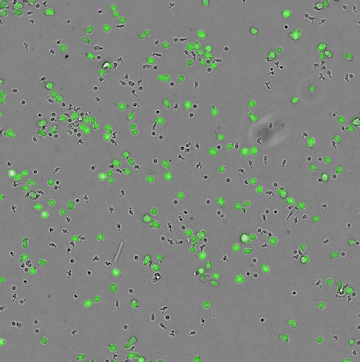


zilovertamab/ IgG1.aF

22.0405.aF

rituximab

**Supplementary Video 1. 22.0405.aF demonstrates fast and potent tumor cell killing.** JeKo-1 target cells stained with CSFE were imaged in the presence of freshly isolated NK cells and 22.0405.aF, zilovertamab, rituximab or isotype control (IgG1.aF) at 50 nM for cell killing under 4 hours using Opera Phenix High Content Screening System. The images are from a presentative experiment from 3 different donors performed in duplicates. The quantification of tumor cell killing is shown in Figure 2E.
